# An Unusual Presentation of *Streptococcus gallolyticus* in Infective Endocarditis

**DOI:** 10.1155/2023/9948719

**Published:** 2023-11-30

**Authors:** Laura Torres Cruz, Maryam Barkhordarian, Neenu Antony, Muhammad Yasir, Sai Priyanka Pulipaka, Ahmad Al-Awwa, Sameh Elias

**Affiliations:** Division of Internal Medicine, Hackensack Meridian Health-Palisades Medical Center, New Jersey, USA

## Abstract

*Background. Streptococcus gallolyticus* (previously known as *Streptococcus bovis* type-1) bacteremia has a well-established, almost pathognomonic association with colorectal carcinoma, with the most common hypothesized mechanism being ulceration of polyps leading to hematologic dissemination. There are few reported cases of streptococcus bacteremia from other, seemingly benign sources like cellulitis or colonic adenomas. Hence, there is limited focus on skin and soft tissue infections leading to potentially fatal infective endocarditis. *Case Presentation.* We present a novel case of streptococcus bacteremia from uncommon sources like abdominal wall cellulitis or colonic adenoma leading to infective endocarditis as well as other manifestations, including osteomyelitis and discitis. This report highlights a unique case of streptococcus bacteremia with an uncommon origin, arising from abdominal wall cellulitis or colonic adenoma, ultimately resulting in the development of infective endocarditis. Furthermore, the patient presented with additional clinical manifestations, including osteomyelitis and discitis. *Conclusions.* Through our case report, we emphasize the importance of investigating uncommon sources like cellulitis when initial malignant workup is negative in streptococcus bacteremia and further elucidate the pathophysiology of streptococcus bacterial dissemination from nonmalignancy-related sources.

## 1. Background

*Streptococcus gallolyticus*, previously known as *Streptococcus bovis* type 1, is a colonizer of human skin in 2-15% of the human population [1]. The correlation between *Streptococcus gallolyticus* bacteremia and colorectal carcinoma has been well established through the adenoma-carcinoma sequence [2]. Although ulceration of a polyp might be one of the pathways leading to bacteremia in patients who are identified to have polyps, it is not clearly known how *Streptococcus gallolyticus* enters into the bloodstream in patients without polyps. We present a case of infective endocarditis with *Streptococcus gallolyticus* bacteremia who presented with abdominal wall cellulitis. The data correlating *Streptococcus gallolyticus* with particularly abdominal wall cellulitis is sparse. Thus, through this case report, we aim to assist clinicians in considering alternative causes of underlying infective endocarditis, such as gastrointestinal diseases, as well as hepatobiliary and pancreatic pathologies, which can help in earlier recognizing and improving patient-centered outcomes.

## 2. Case Presentation

This is a 53-year-old male with a past medical history of uncontrolled type 2 diabetes mellitus who presented for evaluation of one week of new-onset worsening and persistent exertional shortness of breath associated with bilateral leg edema, orthopnea, and paroxysmal nocturnal dyspnea. Three weeks prior, while investigating for abdominal wall cellulitis, blood cultures were positive for *Streptococcus gallolyticu*s, and the patient was found to have infective endocarditis of the aortic and mitral valves. At that time, he was discharged with a ceftriaxone infusion for six weeks.

**Figure 1:**
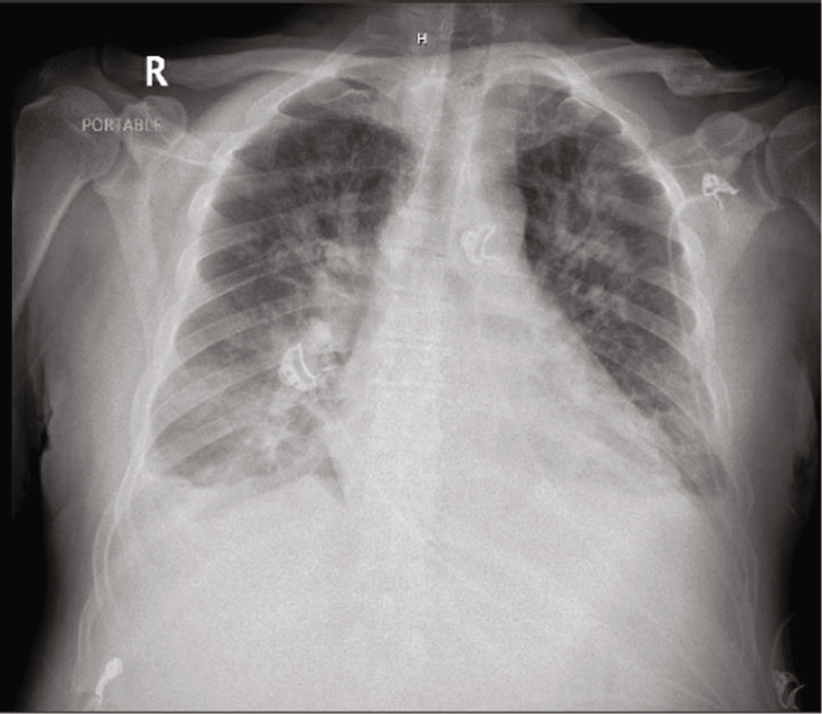
Chest X-ray: multifocal bilateral patchy ground-glass infiltrates with pleural effusions consistent with pulmonary edema.

**Figure 2:**
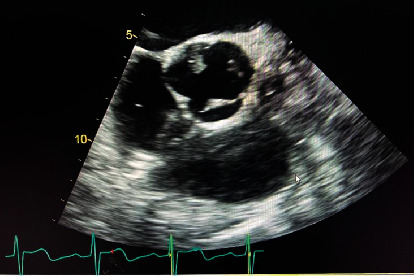
Echocardiogram demonstrating moderate-size aortic valve vegetations in each tricuspid valve leaflet.

**Figure 3:**
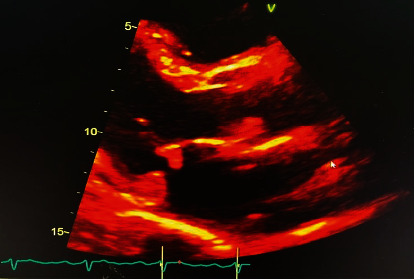
Echocardiogram demonstrating moderate-size aortic valve and moderate-size mitral valve (anterior leaflet) vegetations.

On physical examination, jugular venous distension and hepatojugular reflux were noted; a systolic murmur in the second intercostal space, right parasternal area radiating to the right carotid artery, and a diastolic murmur in the fifth intercostal space of the midaxillary line, bilateral basilar rales, and pitting edema were present. A 2 × 2 cm soft, nontender, immobile mass was appreciated in the right side of the neck. He had a brain natriuretic peptide of 1406 pg/ml (<100) and hemoglobin A1C of 13.6%. The chest X-ray was consistent with pulmonary edema (Figure 1). CT chest revealed a left lower lobe pulmonary embolism and pulmonary edema. The echocardiogram demonstrated a left ventricular ejection fraction of 65-70%, severe aortic regurgitation, and moderate size aortic valve and moderate size mitral (anterior leaflet) valve vegetations, which were larger compared to the previous study three weeks ago (Figures 2 and 3). CT soft tissue neck revealed findings highly concerning for osteomyelitis/discitis at C5/C6 and a prevertebral/retropharyngeal abscess, measuring approximately 7.7 × 2.6 × 5.0 cm in size. The patient was started on furosemide, cultures were repeated, and ceftriaxone was continued.

Due to the severity of valvular involvement, the patient was transferred emergently to our higher level of care facility for aortic and mitral valve replacement. Subsequently, he underwent a C5/C6 corpectomy, C5-6 and C6-7 bilateral discectomy and foraminotomies, placement of vertebral body cage, and cervical plate spanning of C4-C7 and fusion. The repeat blood cultures remained negative for twenty days before valve replacement surgery. Colonoscopy was unremarkable for colon cancer, demonstrating two benign colon polyps consistent with tubular adenoma. The patient was treated for endocarditis for a total of 6 weeks on ceftriaxone 2 grams daily. Following the patient six months after discharge, he has been stable with no active medical issues while following up with the cardiologist.

## 3. Discussion

*Streptococcus gallolyticus*, formerly known as *Streptococcus bovis* type 1, is a gram-positive coccus. It is commonly associated with infective endocarditis, particularly in patients with underlying gastrointestinal pathology and colorectal tumors [3, 4]. It is not clearly elucidated how *Streptococcus gallolyticus* accesses the bloodstream in patients without polyp [2]. One accepted theory is its passage through biliary channels, as *Streptococcus gallolyticus* bacteremia has been identified in cases of acute cholangitis and hepatic abscesses [5]. There is limited data about *Streptococcus gallolyticus* with colonic adenoma [6, 7]. The presence of *Streptococcus gallolyticus* in the colonic adenoma and later mainly found in colorectal cancer demonstrates its possible role in the pathogenesis and oncogenesis in the progression from colonic adenoma to adenocarcinoma [4].

We presented a case of infective endocarditis secondary to *Streptococcus gallolyticus* bacteremia originating from abdominal wall cellulitis. In our case, no association was found between the source of *Streptococcus gallolyticus* and colon cancer. However, we hypothesize that the *Streptococcus gallolyticus*-causing bacteremia and infective endocarditis in our patient were either related to the abdominal wall cellulitis or the benign colonic adenomas. The most common microbes in cellulitis are identified as *Streptococcus pyogenes* and *Staphylococcus aureus* [8]. However, Sacco et al. reported a rare case of *Streptococcus gallolyticus* endocarditis with lower extremities cellulitis and no macroscopic colonic pathology [9]. Therefore, the occurrence of *Streptococcus gallolyticus* as a cause of abdominal wall cellulitis is still possible.

Several studies showed that *Streptococcus gallolyticus* can manifest in conditions beyond infective endocarditis, including osteomyelitis and discitis [10, 11]. Management of pyogenic spondylodiscitis with six weeks of appropriate antibiotics based on the sensitivity was also recommended [12]. The significance of associating *Streptococcus gallolyticus* with not only malignant colonic polyps but also benign polyps should be emphasized, warranting careful monitoring during the management of patients presenting with similar presentation.

## 4. Conclusions

The association of *Streptococcus gallolyticus* bacteremia with colorectal carcinoma is well established in the literature. However, there are only a few reported cases in the literature of *Streptococcus gallolyticus* bacteremia associated with other conditions like abdominal wall cellulitis or benign colonic adenoma resulting in infective endocarditis. This bacterium can also potentially cause life-threatening complications beyond infective endocarditis, such as septic emboli, osteomyelitis, or discitis. This case report highlights an uncommon cause of infective endocarditis secondary to *Streptococcus gallolyticus* and its serious complications. The aim is to broaden the spectrum of possibilities in diagnosing and managing infections caused by these bacteria.

## Data Availability

Data sharing does not apply to this article as no datasets were generated or analyzed during the current study.
